# Description of Variation in Age of Onset of Functional Limitations of Native Hawaiian and Pacific Islanders Compared to Other Racial and Ethnic Groups

**DOI:** 10.3390/ijerph18052445

**Published:** 2021-03-02

**Authors:** Christopher S. Walter, Marie-Rachelle Narcisse, Jennifer L. Vincenzo, Pearl A. McElfish, Holly C. Felix

**Affiliations:** 1 Department of Physical Therapy, University of Arkansas for Medical Sciences, 1125 N. College Ave, Fayetteville, AR 72703, USA; JLVincenzo@uams.edu; 2 Office of Community Health and Research, University of Arkansas for Medical Sciences, 1125 N. College Ave, Fayetteville, AR 72703, USA; narcissem@uams.edu (M.-R.N.); PAMcelfish@uams.edu (P.A.M.); 3 Fay W. Boozman College of Public Health, University of Arkansas for Medical Sciences, Little Rock, AR 72205, USA; FelixHolly@uams.edu

**Keywords:** health disparities, physical function, NATIVE Hawaiian and Pacific Islander (NHPI)

## Abstract

(1) Background: The purpose of this exploratory study was to describe variation in age of onset of functional limitations of Native Hawaiian and Pacific Islanders (NHPI) compared to other racial and ethnic groups. (2) Methods: Adults age 45 years and older who responded to the Functioning and Disability module within the 2014 National Health Interview Survey (NHIS) were included (*n* = 628 NHPI; 7122 non-Hispanic Whites; 1418 Blacks; 470 Asians; and 1216 Hispanic adults). The NHIS Functioning and Disability module included 13 items, which we organized into three domains of functional limitations using factor analysis: Mobility, Gross Motor Skills, and Fine Motor Skills. Responses were summed within each domain. (3) Results: After adjusting for age and sex, we found that racial/ethnic minority groups, with the exception of Asians, experience more functional limitations than Whites. Results further indicate that NHPI adults experienced an earlier surge in all three domains of functional limitations compared to other racial/ethnic groups. (4) Conclusions: These findings are novel and provide additional evidence to the existence of disparities in functional health outcomes across racial/ethnic groups. Future studies are needed to develop targeted and culturally tailored interventions for those most in need.

## 1. Introduction

Rehabilitation professionals are on the front lines of clinical care. As such, they have the opportunity to observe first-hand disparities in health outcomes—particularly functional limitations—of their patients. Existing literature has shown that there is a clear association between age and physical function, such that older individuals are more likely to experience functional limitations compared to younger individuals [1,2]. Racial and ethnic disparities in physical function have been investigated, although studies have focused primarily on Blacks and Whites [3,4], and to a lesser extent, Hispanic racial and ethnic categories [5]. Findings show that minority groups are more likely than Whites to report limitations in mobility and in activities of daily living (ADLs) [6].

Northwest Arkansas is home to a large number of Native Hawaiian and Other Pacific Islanders (NHPI), thus, in our clinical practice, we have observed NHPI community members presenting with functional limitations at younger ages than patients do from other racial/ethnic groups. Onset of functional limitations in the NHPI has not been documented in the literature. This is likely due to the fact that, historically, major US health surveys have grouped NHPI with Asian Americans, thereby masking important differences between these two culturally distinct groups [7]. However, identifying racial and ethnic disparities in health outcomes is important for developing targeted and culturally tailored interventions for those most in need. Therefore, the purpose of this exploratory study was to describe variation in age of onset of functional limitations of Native Hawaiian and Pacific Islanders (NHPI) compared to other racial and ethnic groups [8].

## 2. Materials and Methods

### 2.1. Study Data and Sample

To include multiple racial and ethnic groups in our analyses, we combined two data sources for this study: the 2014 general U.S. National Health Interview Survey (NHIS) with the 2014 NHPI-NHIS [9]. The NHIS is a cross sectional, in-person household interview conducted by the CDC’s National Center for Health Statistics. One adult is randomly selected from each participating household and that person is asked to complete the questionnaire. The NHPI-NHIS was modeled after the general NHIS and was administered to households containing 1 or more NHPI adult residents. We included respondents aged ≥45 years who completed the Functioning and Disability survey module, resulting in a total sample of 10,854 (628 NHPI, 7122 non-Hispanic White, 1418 Black, 470 Asian, and 1216 Hispanic adults). The NHPI sample was restricted to those who reported NHPI as their sole or primary race. The NHPI-NHIS and NHIS used the same questionnaire but different sampling methodologies. Simply combining these two surveys into a single dataset may lead to biased estimates of standard errors. However, Kaminska and Lynn (2017) developed an appropriate method to combine and compare surveys with distinct sampling designs [10], which we were able to apply for this analysis. First, a stratum indicator that reflects the sampling strata from each survey was created. Stratum identifications were verified to remain unique after combining the two datasets. Because both surveys used a multi-stage design, a primary sampling unit (PSU) indicator was created to reflect multi-stage design with a unique value for each PSU. The uniqueness of the PSUs was verified after combining both datasets. Sampling weights and variance estimation variables were taken into account; and, confirmation that the point estimates had not changed after combining datasets was established. For all variables, misspecification effect—which is the ratio of the true variance of a sample statistic under the complex sample design to the estimated variance when ignoring all or part of the sample design [10]—was not found. Specifications indicated by the National Center for Health Statistics (NCHS) regarding the use of sampling weights and the steps delineated by Kaminska and Lynn (2017) were followed to combine the datasets and analyze the data in an unbiased way [10,11]. Thus, results from this study can be generalized to the population of civilian, non-institutionalized adults (NHPI, non-Hispanic Whites, Blacks, Asians, and Hispanics) aged 45 years and older. Rather than excluding participants, we created a study subpopulation for the estimation. We used STATA/SE 16 for all analyses, which has features for design-based analysis of subpopulation analysis for complex-sample survey data [12]. The NHPI-NHIS and general NHIS contain de-identified public data. Therefore, participant consent was not needed and the Institutional Review Board considered the study exempt.

### 2.2. Measures

The NHIS Functioning and Disability module included 13 items, which we organized into three domains using factor analysis and scree plots. The first domain—Mobility—grouped seven items related to assistance needed for mobility. The second domain—Gross Motor Skills (GMS)—grouped three items related to walking. The third domain—Fine Motor Skills (FMS)—grouped three items related to self-care and use of hands/arms. Response options of Refused/Not ascertained/Don’t know were set as missing (<5% for any item). Other narrative response options were recoded as numeric values and summed to create three functional limitation scales. The Mobility domain scale ranged from 0 to 7, while the scales for the other two domains ranged from 0 to 9. Higher scores indicated more difficulties for all three scales. See Table 1 for specific items and response options. Age was measured in five-year age groups starting at age 45. Sex was female versus male.

### 2.3. Analysis

We used summary statistics to describe functional limitations overall and by race/ethnicity, age and sex groups, and used ordinary-least-squares regression to examine variation in functional limitation by race/ethnicity group while controlling for age and sex. We included an interaction term (race/ethnicity × age groups) to investigate the age heterogeneity effect. We calculated marginal effects and graphed estimated means to visualize the interaction. Post hoc diagnostic tests revealed that the models did not violate linearity and normality assumptions.

## 3. Results

The weighted average age of respondents was 60.7 (0.15) and 52.8% of respondents were female. Summary statistics describing overall functional limitations, and by race/ethnicity, age and sex groups, are presented in Table 2.

The regression model indicated significant variation in functional limitation scores by race/ethnicity (see Table 3). NHPI and Blacks have significantly higher Mobility scores than Whites (β = 0.11, SE = 0.05; β = 0.13, SE = 0.03, respectively). All of the race/ethnicity groups had significantly different GMS scores than Whites but the results were mixed: NHPI (β = 0.26, SE = 0.09), Blacks (β = 0.41, SE = 0.07), and Hispanics (β = 0.21, SE = 0.07) had significantly higher GMS scores than Whites, but Asians had significantly lower scores than Whites (β = −0.31, SE = 0.08). NHPIs (β = 0.11, SE = 0.05), Blacks (β = 0.09, SE = 0.03), and Hispanics (β = 0.17, SE = 0.04) had significantly higher FMS scores than Whites. There was no difference in scores between Asians and Whites.

**Figure 1:**
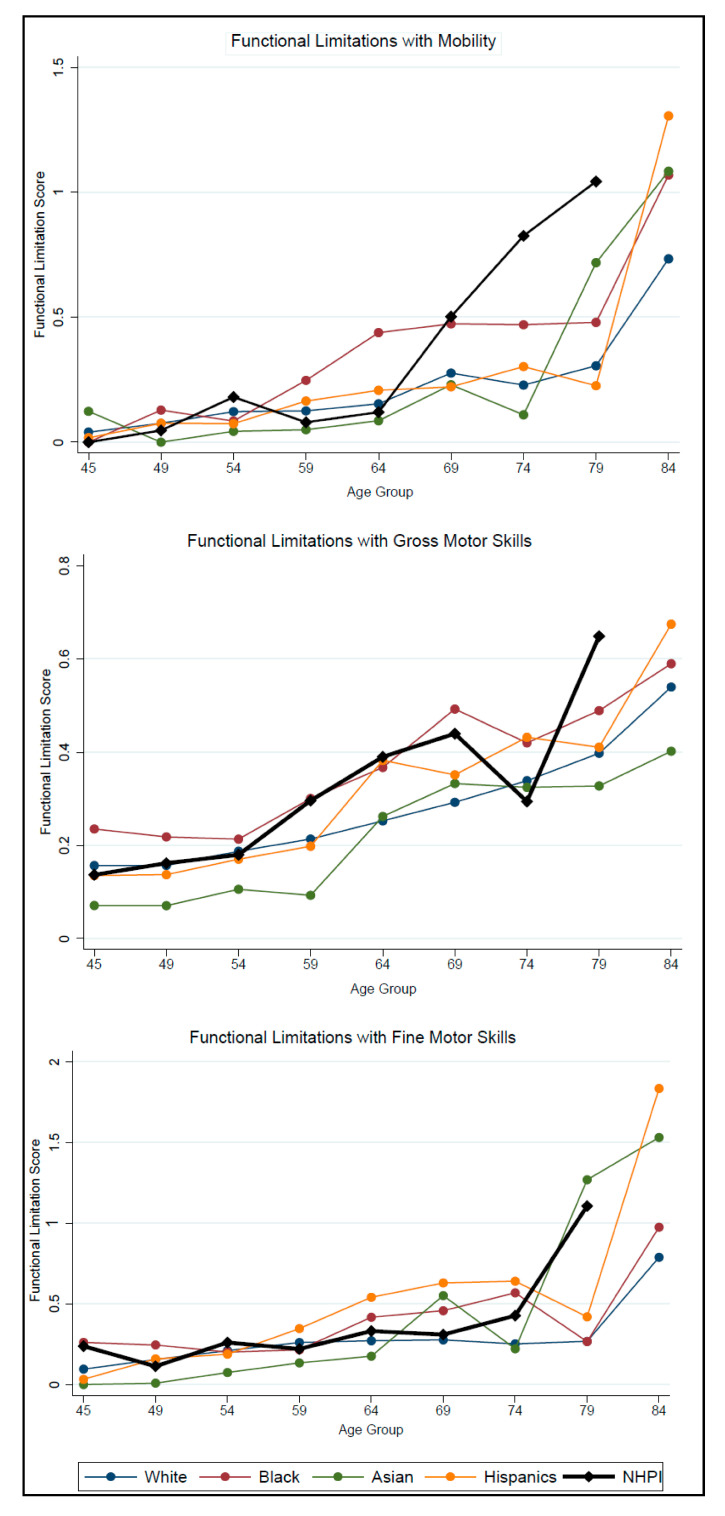
Showing the functional limitations by race/ethnicity and age group for Mobility, Gross Motor Skills (GMS), and Fine Motor Skills (FMS), respectively. Higher scores indicate more difficulties for all three functional limitation domains. Note: NHPI = Native Hawaiian and Pacific Islanders.

Figure 1 illustrates the variation in functional limitations by race/ethnicity and age. Across the three domains, the level of functional limitations remained relatively similar for all race/ethnicity groups across the younger age groups, with limitations surging in the later age groups. NHPI adults experienced an earlier surge in mobility limitations, with limitations increasing sharply around age 64, while those of other race/ethnicity groups did not experience a surge for another decade or more. For both GMS and FMS limitations, NHPI adults experienced a surge around age 74, while adults of other race/ethnicity groups did not experience a surge until age 79. The exception was that Asian adults also experienced a surge in FMS limitations at around age 74.

## 4. Discussion

This study describes variation in functional limitations among US adults aged ≥45 years by race/ethnicity. To ensure that we captured the onset of functional limitations, we chose to include middle-aged adults in our analyses. A delimitation of this approach was that it skewed the average age younger (M = 60.7; SD = 0.16 years old). This is likely the reason why our average scores across the three domains of functional limitations were relatively small. Nevertheless, the results are clinically relevant as they show the age of onset of functional limitations by racial group, which could provide a target age for interventions to begin to delay the surges in functional limitations we observed. 

After adjusting for age and sex, we found that racial/ethnic minority groups, with the exception of Asians, experience more functional limitations than Whites. Although previous studies have found similar results [5,6], this article adds new knowledge as the first study to examine racial/ethnic variation in the onset of functional limitations across three domains of functional limitations. Furthermore, this is the first study to include NHPI disaggregated from Asians and demonstrates that NHPI populations report functional limitations at an earlier age than adults from other racial/ethnic groups—consistent with our clinical observations. What is not clear, however, is why NHPI adults experience an earlier onset of functional limitations. The NHPI are disproportionately affected by chronic diseases. NHPI adults have a higher rate of coronary heart disease, angina, and history of heart attack compared to White adults [13]. The prevalence of overweight and obesity is consistently higher among NHPI compared to other ethnic groups [14]. Self-reported diagnosis of diabetes among NHPI range from 12 to 19.1% compared to 9.4% in the general population [15]. Some subgroups of NPHI report upwards to 40% with diabetes [16,17]. To compound these high disease rates, the NHPI have poor self-care behaviors [18,19] and also have limited access to healthcare compared to the general population [20]. These factors, collectively, may have serious implications for mobility and function, which may contribute to their earlier onset of functional limitations. Given that the NHPI is the second fastest growing population in the U.S. [21], their healthcare needs are expected to grow. The early onset of functional limitations found in the NHPI compared to other racial/ethnic minorities should facilitate early and progressive treatment to mitigate functional decline in middle and older aged NHPI individuals.

## 5. Conclusions

This analysis was exploratory and descriptive in nature, allowing us to focus on describing the functional levels and differences between race/ethnic groups. Therefore, results should be interpreted cautiously as study limitations exist. For example, results of this study are limited by not including other factors (e.g., presence of chronic diseases, body mass index, wealth, and level of education [22], etc.) that may play a role in physical functioning. Further studies should incorporate these additional factors to provide a better understanding of racial/ethnic variation in onset of functional limitations, specifically the determinant of early onset of functional limitations among NHPI adults. These results are also limited by the cross-sectional nature of the data and self-reports of functioning. There is evidence to suggest that different cultures react to physical conditions differently [23], thus respondents may have reported perceptual limitations rather than actual limitations. To mitigate perceptual differences across culture and to control for other biases associated with self-report outcomes [24,25], additional studies using objective measures of physical function are needed. Despite these limitations, these results highlight the importance of desegregating NHPI from Asian Americans. They also begin to highlight disparities in functional health outcomes in the most vulnerable populations. Additional research to improve our understanding of these disparities—and how they interact clinically—is critical to designing and implementing evidence-based programs and health policies that will improve the health of our communities.

## Figures and Tables

**Table 1:** Functional Limitation Domains.

Mobility: Do You Use…
A cane or walking stick?
Walker or zimmer frame?
Crutches?
Wheelchair or scooter?
Artificial limb?
Someone’s assistance?
Some other type of equipment or help?
Item responses were coded as Yes = 1; No = 0
Cronbach’s alpha = 0.88
Gross Motor Skills: Do you have difficulty walking…
100 yards on level ground, that would be about the length of one football field or one city block?
a third of a mile on level ground, that would be the length of five football fields or five city blocks?
up or down 12 steps?
Item responses were coded as No difficulty = 0; Some difficulty = 1; A lot of difficulty = 2; Cannot do at all/Unable to do = 3
Cronbach’s alpha = 0.83
Fine Motor Skills: Do you have difficulty…
with self-care, such as washing all over or dressing?
raising a 2 L bottle of water or soda from waist to eye level?
using your hands and fingers, such as picking up small objects, for example, a button or pencil, or opening or closing containers or bottles?
Item responses coded as No difficulty = 0; Some difficulty = 1; A lot of difficulty = 2; Cannot do at all/Unable to do = 3
Cronbach alpha = 0.70

**Table 2:** Functional Limitation Distribution across Race/Ethnicity, Age, and Sex.

	Use of Assistance	Difficulty Walking	Difficulty with Performing Self-Care Activities
Study population	0.23 (0.01)	0.96 (0.02)	0.32 (0.01)
Race/Ethnicity			
*White*	0.22 (0.01)	0.92 (0.03)	0.29 (0.01)
*Black*	0.33 (0.03)	1.25 (0.07)	0.35 (0.03)
*Asian*	0.19 (0.05)	0.56 (0.08)	0.34 (0.34)
*Hispanic*	0.20 (0.02)	0.98 (0.07)	0.41 (0.04)
*NHPI*	0.27 (0.06)	1.01 (0.10)	0.32 (0.05)
Age			
*45–49*	0.08 (0.02)	0.48 (0.04)	0.17 (0.02)
*50–54*	0.11 (0.02)	0.64 (0.05)	0.20 (0.03)
*55–59*	0.15 (0.02)	0.74 (0.05)	0.27 (0.03)
*60–64*	0.18 (0.02)	0.96 (0.06)	0.31 (0.03)
*65–69*	0.29 (0.03)	1.11 (0.07)	0.34 (0.04)
*70–74*	0.27 (0.03)	1.25 (0.09)	0.33 (0.04)
*75–79*	0.33 (0.05)	1.33 (0.10)	0.31 (0.04)
*80–84*	0.58 (0.07)	1.88 (0.13)	0.64 (0.09)
*85 years+*	1.05 (0.09)	2.59 (0.13)	1.18 (0.12)
Sex			
*Male*	0.19 (0.01)	0.73 (0.03)	0.23 (0.02)
*Female*	0.27 (0.02)	1.17 (0.04)	0.40 (0.02)

Source: National Health Interview Survey (NHIS), 2014 and Native Hawaiian and Other Pacific Islanders—NHIS, 2014.

**Table 3:** Associations between Functional Limitations and Race/Ethnicity.

	β Estimates	Standard Error	*p*-Values	95% Confidence Interval	95% Confidence Interval
				Lower Bound	Upper Bound
Use of Assistance					
*Black*	**0.13**	0.03	<0.001	0.07	0.2
*Asian*	−0.01	0.04	0.849	−0.09	0.07
*Hispanic*	0.03	0.02	0.144	−0.01	0.08
*NHPI*	**0.11**	0.05	0.036	0.01	0.22
Difficulty walking					
*Black*	**0.41**	0.07	<0.001	0.28	0.54
*Asian*	**−0.31**	0.08	<0.001	−0.46	−0.15
*Hispanic*	**0.21**	0.07	0.002	0.08	0.34
*NHPI*	**0.26**	0.09	0.007	0.07	0.44
Difficulty with performing self-care activities					
*Black*	**0.09**	0.03	0.006	0.02	0.15
*Asian*	0.05	0.06	0.340	−0.06	0.17
*Hispanic*	**0.17**	0.04	<0.001	0.09	0.24
*NHPI*	**0.11**	0.05	0.037	0.01	0.21

Source: National Health Interview Survey (NHIS), 2014 and Native Hawaiian and Other Pacific Islanders—NHIS, 2014. Note: White is the reference category. Estimates with *p* < 0.05 are bolded. Models associated for age and sex.

## Data Availability

Publically available datasets were analyzed in this study. This data can be found here: https://www.cdc.gov/nchs/nhis/nhis_questionnaires.htm and https://www.cdc.gov/nchs/nhis/nhpi.html.
